# French Ministry of Health’s response to Paris attacks of 13 November 2015

**DOI:** 10.1186/s13054-016-1259-8

**Published:** 2016-04-01

**Authors:** Jean-Marc Philippe, Olivier Brahic, Pierre Carli, Jean-Pierre Tourtier, Bruno Riou, Benoit Vallet

**Affiliations:** Health Emergencies Crisis Management Centre, French Ministry of Health, 14 Avenue Duquesne, 75007 Paris, France; Paris Health Emergency Units (SAMU), 149 rue de Sèvres, 75015 Paris, France; Emergency Services Paris Fire Brigade (BSPP), 3 boulevard de l’Yser, 75017 Paris, France; Emergency Services of “Pitié-Salpêtrière” Hospital, 83 boulevard de l’Hôpital, 75013 Paris, France; General Diretorate for Health, French Ministry of Health, 14 avenue Duquense, 75007 Paris, France

## Abstract

On Friday November 13th at 9:20 pm, three kamikaze bombs went off around the Stade de France a stadium in Saint-Denis just outside Paris, 4 different shootings took place and bombings in Paris and hundreds of people were held hostage in a theater.

This multi-site terrorist attack was the first of this magnitude in France. Drawing the lessons of these attacks and those which occurred in other countries from a health perspective is essential to continuously adapt and improve the French response to possible future attacks.

Several issues would need to be further explored:

- Management of uncertainties: When to trigger the plans: after the 1st attack, the 2nd? When do attacks end and when to release mobilized resources?
- Management of victims: How to ensure that all victims are secured or taken care of? How to provide assistance when attacks are ongoing?
- Management of teams: Proper follow-up of persons involved in the response: health professionals, police and firemen, emergency call centers but also civil servants within administration that contributed to the response.
- Communication: Reactivity of all is a key element to secure appropriate resource is mobilized for the response. All actors have to be able to communicate quickly in a secured way.

On Friday, 13 November 2015, at 9:20 p.m., three suicide bombers struck near the Stade de France, a stadium in Saint-Denis just outside Paris; four different shootings took place; and hundreds of people were held hostage in a theatre. This multi-site terrorist attack was the first of this magnitude in France.

In the event of multi-site attacks, France activates its emergency plans. An interministerial crisis centre (ICC) under the authority of the prime minister is activated to coordinate actions and mobilisation of various actors. As regards the Ministry of Health, the Health Emergencies Crisis Management Centre (HECMC) is installed within the permanent centre for operational reception and regulation of health emergencies and then is activated.

The role of the HECMC is the following:

- to ensure national coordination of the health-care system;
- to analyse and follow up the situation on the basis of the information provided by regional health agencies and provide a regular update on the situation at the national level; and
- to represent the Ministry of Health within the ICC and take part in the response organised by this centre.

The emergency plan aims to provide an adapted response and mobilisation of resources. The main elements of the plans are the following:

- mobilisation of health professionals through the “Hospital Mobilization Plan” called “white plan”;
- no accumulation of resources on the first site, to optimise management of resources in case of multiple attacks;
- each site has its own emergency unit team and evacuation system;
- distribution of resources according to predefined intervention zones;
- mobilisation of receiving hospitals and other resources (emergency units and hospitals) to back up mobilised resources;
- simplified treatment of victims according to the pathologies (head, thorax, abdomen, orthopaedics, blast or paediatrics); and
- triage of victims: absolute emergencies and relative emergencies.

After the “Charlie Hebdo” shootings on 7 January 2015, emergency departments in and out of the hospital undertook simulation exercises. Feedback on attacks which occurred in other countries in the past [1, 2] has been incorporated in the reflection on and preparation for these exercises. Several drills had been organised, the last of which took place at 10 o’clock on the morning of 13 November, but nothing could prepare the medical teams for the violence observed that night.

As early as 9:30 p.m., the HECMC was installed by the Director General for Health [3]. In the minutes after the shooting, eight French Health Emergency Units (SAMU) of the Paris region and the Paris Fire Brigade (BSPP) deployed more than 40 medical teams to the different sites.

The HECMC rapidly mobilised the following:

- the Regional Health Agency of the Paris Region to ensure a permanent follow-up of the situation and organise the care of victims within the Paris Region hospitals (17 hospitals of Assistance Publique-Hôpitaux de Paris, two military hospitals and 25 other hospitals) but also to seek assistance for the emergency ambulances;
- the peripheral regional health agencies as back-up resources;
- emergency psycho-social support units (EPSSUs) to provide support to victims or their relatives or both;
- the French Blood Agency to supply hospitals with an appropriate level of blood; and
- the Health Emergency Preparedness and Response Agency (EPRUS) in case additional resources would have been needed.

Over the weekend, the HECMC organised 12 phone conferences, including one dedicated to the management of families and victims. It consolidated the list of victims in liaison with the Paris Regional Health Agency, Paris Hospitals and Army Health Services for transmission to the ICC.

On 14 November, the HECMC coordinated the organisation of psychological support to victims and families. EPSSUs were installed in Paris District town halls as well as within the Military School and the Legal and Forensic Medicine Institute. EPSSUs from other departments were also involved.

That night, 124 people died. Six hundred and forty-three injured people were taken care of in Paris Region hospitals. Seven patients died in a hospital setting. Thirty-seven patients were in the intensive care unit (ICU) at the Assistance Publique-Hôpitaux de Paris, and five in other ICUs. Seven hospitals activated more than 30 surgical teams and operating rooms in the middle of the night [4].

Eight hundred and eighty-six medico-psychological consultations were held in Paris town halls of the 10th and 11th districts, 567 consultations at the Military School, and 465 consultations at the Legal and Forensic Medicine Institute. Additionally, 230 consultations were held at the Hotel-Dieu Hospital, which served as the main site where associations were providing psychological support to victims.

The Paris attacks ultimately killed 130 persons.

Drawing the lessons of these attacks and those which occurred in other countries [1, 2] from a health perspective is essential to continually adapt and improve the French response to possible attacks in the future.

Several issues need to be further explored:

- Management of uncertainties: When to deploy the plans: after the first attack, the second? When do attacks end and when to release mobilised resources?
- Management of victims: How to ensure that all victims are secured or taken care of? How to provide assistance when attacks are ongoing?
- Management of teams: Proper follow-up of persons involved in the response: health professionals, police and fire personnel, emergency call centres but also civil servants within the administration who contributed to the response.
- Communication: Responsiveness is a key element to ensure that appropriate resources are mobilised for the response. All actors have to be able to communicate quickly in a secured way.

## Abbreviations

EPSSU: Emergency psycho-social support unit
HECMC: Health Emergencies Crisis Management Centre
ICC: Interministerial crisis centre
ICU: Intensive care unit
